# Applications of Nanozymes in Chiral-Molecule Recognition through Electrochemical and Ultraviolet–Visible Analysis

**DOI:** 10.3390/molecules29143376

**Published:** 2024-07-18

**Authors:** Jing-Jing Dai, Guo-Ying Chen, Lei Xu, Huan Zhu, Feng-Qing Yang

**Affiliations:** School of Chemistry and Chemical Engineering, Chongqing University, Chongqing 401331, China; 202318021129t@stu.cqu.edu.cn (J.-J.D.); 20221801017@stu.cqu.edu.cn (G.-Y.C.); 20195277@cqu.edu.cn (L.X.); 202318021131t@stu.cqu.edu.cn (H.Z.)

**Keywords:** nanozyme, enantioselectivity, chiral-molecule, ultraviolet–visible spectrometry, electrochemical analysis

## Abstract

Chiral molecules have similar physicochemical properties, which are different in terms of physiological activities and toxicities, rendering their differentiation and recognition highly significant. Nanozymes, which are nanomaterials with inherent enzyme-like activities, have garnered significant interest owing to their high cost-effectiveness, enhanced stability, and straightforward synthesis. However, constructing nanozymes with high activity and enantioselectivity remains a significant challenge. This review briefly introduces the synthesis methods of chiral nanozymes and systematically summarizes the latest research progress in enantioselective recognition of chiral molecules based on electrochemical methods and ultraviolet–visible absorption spectroscopy. Moreover, the challenges and development trends in developing enantioselective nanozymes are discussed. It is expected that this review will provide new ideas for the design of multifunctional chiral nanozymes and broaden the application field of nanozymes.

## 1. Introduction

Chirality refers to the property of an object that lacks geometric symmetry with its mirror image, akin to the left hand and the right hand, which are mirror images of each other but cannot be superimposed [1]. Chirality is closely related to life activities; for example, most natural amino acids are levorotatory (L-configuration) except glycine, and the double helix of DNA molecules is predominantly right-handed helix [2]. In addition, neurotransmitters, alkaloids, carriers, hormones, and other compounds closely related to body functions are chiral compounds [3]. It is noteworthy that although enantiomers of chiral molecules have many similar physical and chemical properties, they often have significant differences in physiological activity and toxicity. For example, one enantiomer of a chiral drug is the active ingredient for treatment, but the other enantiomer is useless or even produces toxic side effects; the “thalidomide tragedy” is a typical example [4]. Currently, more than 50% of drugs, 40% of agrochemicals, and about 30% of pesticides are chiral, and most of them exist in the form of racemates or mixtures [5,6]. However, the use of single enantiomers will have higher safety and better effects. Therefore, the separation and selective recognition of enantiomers are crucial for medicine, the environment, and life sciences.

There are several techniques traditionally used in chiral recognition, such as capillary electrophoresis [7], gas chromatography [8], high-performance liquid chromatography [9], nuclear magnetic resonance [10], etc. Although the above methods have high reliability and sensitivity, the testing conditions are complex, expensive, and laborious. In recent years, electrochemical and optical technologies have become effective means of chiral recognition due to their advantages of easy operation, low cost, real-time analysis, high sensitivity, and visual discrimination [11]. It is well known that chiral recognition in living organisms mainly relies on the specificity of natural enzymes. For example, glucose oxidase (GOx) can only catalyze the oxidation of D-glucose (D-Glu), while it remains inert to L-glucose (L-Glu). D-amino acid oxidase selectively catalyzes the oxidation of D-amino acids rather than L-amino acids [12,13]. The efficiency and specificity of natural enzymes for chiral molecules, when combined with electrochemical and optical techniques, are conducive to enhancing their chiral recognition ability towards enantiomers [14]. For example, Yi et al. [15] prepared a nitrogen-doped silicon quantum dots/silver (N-SiQD/Ag NPs) probe and coupled it with β-D-glucose oxidase to directly identify Glu enantiomers using colorimetric and fluorescent dual signals. A good linear relationship was obtained with D-Glu concentration of 1.0–200.0 μM and a limit of detection (LOD) of 50 nM.

It is worth noting that the nanomaterials have great potential in mimicking the catalytic performance and stereoselectivity of natural enzymes [16]. Compared with natural enzymes, nanomaterials with enzyme-like activities (nanozymes) have the unique advantages of low cost, adjustable activity, and high stability under harsh conditions, which can avoid the disadvantages of high cost, easy deactivation, and difficulty in large-scale production of natural enzymes [17]. Since Fe_3_O_4_ nanoparticles (Fe_3_O_4_ NPs) have been proven to have intrinsic peroxidase (POD)-like activity, many nanozymes have been reported, including metal-based nanozymes, carbon-based nanozymes, and emerging single-atom nanozymes [18]. The enzyme-like activities include POD, superoxide dismutase (SOD), oxidase (OXD), catalase (CAT), hydrolase, etc. [19] Although nanozymes have made great progress in various fields, such as biosensing, disease treatment, and environmental protection, it is difficult to achieve selective recognition and catalysis of enantiomers in most cases, due to the lack of fine surface structures, charges, and hydrophilic/hydrophobic binding sites [20]. Most related research has only focused on improving the catalytic efficiency of nanozymes, causing the stereoselectivity of nanozymes to not yet receive enough attention. Therefore, it is crucial to rationally construct and synthesize nanozymes with specific recognition pockets and stereoselectivity for the recognition of chiral substances. For example, Li et al. [21] synthesized chiral copper oxide flowers (S-CuO) with POD-like activity through a one-pot method. Due to the helical arrangement of subnanopetals in a specific pattern in S-CuO, it exhibits a greater affinity for S-mandelic acid (S-MA) than R-MA, thus achieving enantioselective recognition of R/S-MA. Zhu et al. [22] integrated AuNPs with OXD-like activity and chiral Cu–metal–organic frameworks onto TiO nanotube arrays to fabricate a homochiral light-sensitive organic photoelectrochemical transistor (OPECT), which was applied for the enantioselective recognition of D/L-Glu. The linear dynamic range for D/L-Glu was 0.1–10 μM and 10–10000 μM, with LOD values of 0.05 μM and 0.07 μM, respectively.

Nanozymes are potential candidates to be applied in the highly sensitive and enantiomer -selective analysis of biological and environmental samples. First, this review briefly introduces the synthesis methods of chiral nanozymes and then delves into the current state of research advancements concerning the enantioselective catalysis and identification of chiral compounds, leveraging nanozymes-empowered electrochemical techniques and ultraviolet–visible (UV–Vis) spectroscopy. In addition, the challenges and future prospects of nanozymes in the field of chiral recognition are also discussed. This review may provide a reference for future research on the design and application of stereoselective nanozymes, with the hope of expanding the research and application of nanozymes and further narrowing the gap between nanozymes and natural enzymes for chiral recognition.

## 2. Synthesis of Chiral Nanozymes

Chiral interactions between the chiral sites of enzymes and substrates are the basis for enantioselective recognition. To improve the enantioselectivity of nanozymes, the synthesis of chiral nanozymes is of significant importance. The chirality of nanozymes is closely related to their chiral ligands, shapes, and crystal structures. In general, the main methods to synthesize chiral nanozymes are (1) functionalization of nanozymes by chiral ligands [23,24,25], (2) intrinsic chirality formed by lattice distortion or defects of nanozymes induced by chiral ligands [21,26,27], and (3) self-assembly of chiral templates [28,29,30].

Using chiral ligands to modify and functionalize nanozymes is the most commonly used method for the synthesis of chiral nanozymes, in which the introduction of chirality mainly relies on the chiral interactions between the nanozymes and the chiral ligands. Among the chiral ligands, chiral amino acids, peptides, DNA, and chiral metal complexes are the most common choices [31]. Most of the natural amino acids, except for glycine, are chiral, and chiral amino acid residues are essential in the enantioselective recognition of natural enzymes [32,33]. Therefore, using chiral amino acids as ligands has led to the construction of various chiral nanozymes inspired by the structure of natural enzymes. The chiral amino acids not only provide chiral sites and confer enantioselectivity to the nanozymes, but also serve as reducing agents and stabilizers, reducing metal salts for the preparation of nanomaterials. Meanwhile, the modification of nanozymes with amino acids, which encapsulate the surface of the nanozymes, can enhance their stability. For example, Zhang et al. [34] prepared nanoflower-like MoS_2_/CoS_2_ NPs capped with D/L-cysteine (D/L-Cys). They first synthesized MoS_2_/CoS_2_ NPs using the classical hydrothermal method, and then modified their surface through the interaction between the thiol groups of D/L-Cys and MoS_2_/CoS_2_ NPs to obtain chiral nanozymes (D/L-NPs). The circular dichroism spectrum of D/L-NPs showed mirror symmetry, indicating the successful preparation of the chiral nanozymes. Moreover, the modification with D/L-Cys restricted the aggregation of MoS_2_/CoS_2_ NPs, enhancing their dispersion stability and effectively improving the catalytic performance of D/L-NPs. For metal and metal-oxide-based nanomaterials, chiral nanozymes can also be constructed by surface modification using chiral compound molecules [35]. For instance, Prins et al. [36] reported the chiral nanozyme [TACN·Zn^2+^]-AuNPs (named (+)−1 and (+)−1), which were prepared through modifying AuNPs with thiocyanate triazacyclononane (TACN) Zn^2+^ of different configurations, self-assembling on the surface of bis(octylamine)-passivated AuNPs to form the chiral nanozyme. Circular dichroism spectroscopy measurements showed two opposite signals, confirming the presence of enantiomeric thiols on the nanozyme surface, forming an active catalytic pocket, and the chirality originated from the D/L-serine (D/L-Ser) in the head group of the nanozyme. The chiral nanozyme synthesized by this strategy exhibits nuclease-like catalytic activity that can catalyze the cleavage of phosphodiester bonds and promote transphosphorylation reactions, showing different reactivity towards enantiomers of pure RNA model substrates.

The intrinsic chirality of nanomaterials primarily originates from lattice distortions or defects in the crystal structure, which give rise to the intrinsic chirality of the nanostructure [37]. During the synthesis of nanozymes, the addition of chiral inducers can guide the growth direction of the lattice or cause lattice distortions, leading to the formation of chiral lattices with uniform rotational stacking in the nanocrystals [38]. For example, Li et al. [39] synthesized chiral Au nanocrystals with chiral morphology and more exposed high-refractive-index facets using a chiral inducer (D/L-Cys). Initially, KBr, KI, and N-hexadecyltrimethylammonium chloride were used together as morphology inducers to accelerate the reduction of Au^3+^ and crystal growth. Then, D/L-Cys was introduced as a chiral inducer to synthesize chiral Au nanocrystals (D/L-Cys-Au). The thiol group of Cys binding with Au leads to a slower crystal growth rate at the binding sites, causing asymmetrically structural evolution of the Au nanocrystals. Notably, the structural evolution induced by D-Cys and L-Cys are counterclockwise and clockwise spiral structures, respectively. Circular dichroism spectroscopy indicates that the chirality of D/L-Cys-Au originates from the chiral morphology of D/L-Cys-Au rather than the chirality of the D/L-Cys molecules on the surface of Au nanocrystals. Li et al. [40] also synthesized nanozymes with chiral morphology (D/L-Cys@Au/Fe Hops) using chiral Cys molecules as inducers through a simple solvothermal method. The nanopetals of L-Cys@Au/Fe Hops exhibit a right-handed helical orientation, while those of D-Cys@Au/Fe Hops exhibit a left-handed helical orientation. The chiral morphology endows D/L-Cys@Au/Fe Hops with high enantioselectivity, achieving chiral recognition of D/L-3,4-dihydroxy-phenylalanine (D/L-DOPA).

Chiral templating self-assembly offers a powerful tool for obtaining chiral structures, and different chiral templates can produce a variety of chiral self-assembled materials. Chiral nanozymes can be rationally designed and constructed through the efficient self-assembly of peptides, amphiphilic compounds, chiral surfactants, and so on. Peptides are formed by the dehydration condensation of amino acids and driven by noncovalent interactions such as hydrophobic interactions, electrostatic interactions, π-stacking, and hydrogen bonding; they readily self-assemble into various nanostructures [41]. Chiral nanozymes obtained through peptide self-assembly not only possess chiral atoms but also provide chiral spatial structures that create an environment conducive to selective catalysis [42]. For example, Wang et al. [43] assembled a chiral nanozyme system containing glutamine peptide (Q peptide), heme, and G-quadruplex DNA (G-DNA). G-DNA serves as an axial ligand and supramolecular scaffold that can effectively bind to heme through π-interactions and axial coordination. The Q peptide can self-assemble into β-sheet fibrous nanostructures in a saline environment. The intermolecular association of the Gln peptide may lead to the local enrichment and orientation of the carboxyl amide, thereby providing potential multivalent hydrogen bonds and enhancing the affinity of H_2_O_2_ for heme. Consequently, the β-sheet forming ability of the Q peptide significantly affects the catalytic synergy between G-DNA and the peptide. Furthermore, the self-assembly of the Q peptide with the G-DNA/hemin DNAzyme exhibits enantioselectivity towards D/L-DOPA. This modular self-assembly strategy paves the way for creating a chiral environment.

## 3. UV–Vis Spectroscopy

Optical methods, especially those based on UV–Vis absorption spectroscopy and fluorescence spectroscopy, have garnered significant attention due to their high sensitivity, real-time analysis capabilities, low cost, and diverse signal output modes [44]. As shown in Table 1, UV–Vis spectroscopy enhanced by chiral nanozymes is commonly used for the enantioselective recognition of chiral molecules, including D/L-DOPA, D/L-tyrosine (D/L-Tyr), D/L-Glu, D/L-Tyrosinol, etc. Nanozymes can catalyze chiral substrates to produce products with distinct absorption peaks in the UV–Vis spectra. Due to the stereoselectivity of nanozymes, they will exhibit different catalytic efficiencies towards two enantiomers. Consequently, chiral-molecule recognition can be attained by leveraging the disparities in the absorption peaks of the products.

Different novel nanomaterials were designed to mimic the principles of natural enzymes to reproduce their catalytic activities [61]. However, endowing nanozymes with stereospecificity remains a challenge. Considered as physical analogs of extracellular matrix components, carbon nanomaterials are deemed an excellent choice for the development of nanozymes [62]. In 2010, Qu’s team first reported that carboxyl-modified graphene oxide (GO-COOH) possesses intrinsic POD-like activity [63]. Interestingly, the modification of the GO-COOH surface with zinc finger protein α-helical chiral metal supramolecular complex ([Fe_2_L_3_]^4+^) showed a higher POD-like activity [45]. [Fe_2_L_3_]^4+^ consists of left-handed spiral [Fe_2_L_3_]^4+^ (Fe-M) and right-handed spiral [Fe_2_L_3_]^4+^ (Fe-P), and Fe-P-GO-COOH shows higher catalytic activity than Fe-M-GO-COOH, demonstrating enantioselective catalytic activity that can be used for distinguishing chiral drugs (D/L-DOPA). However, the discrimination mechanism is still unclear and warrants further exploration.

**Figure 1:**
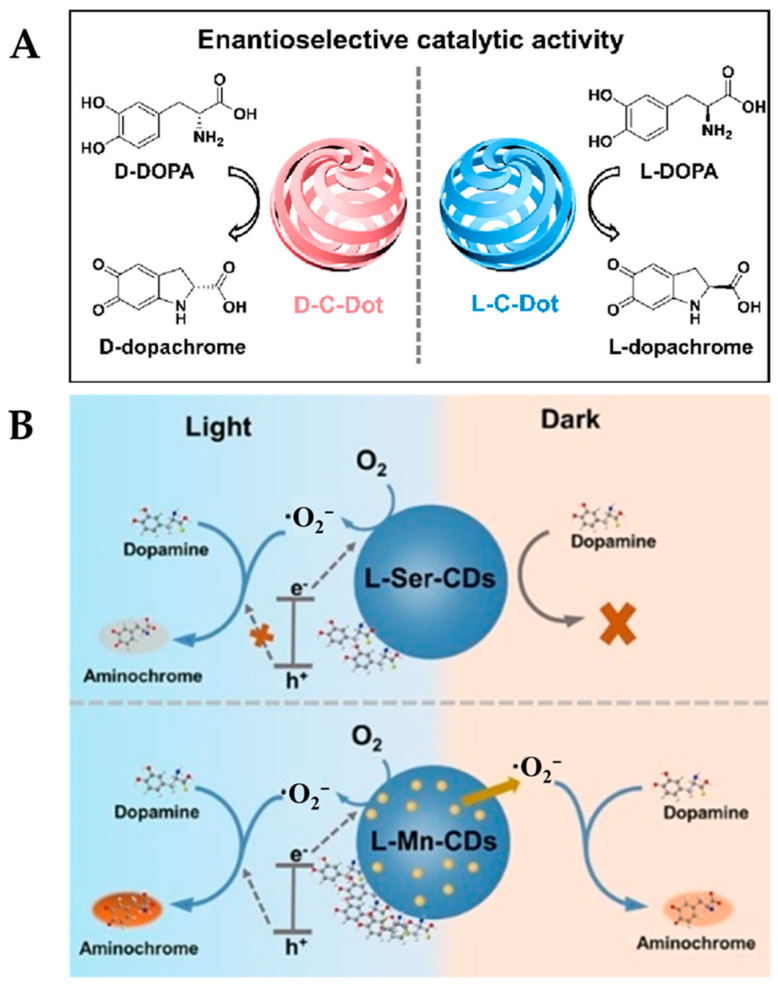
(**A**) Schematic representation of the enantioselective catalytic activity of D/L-C-Dots for the oxidation of D/L-DOPA [26] and (**B**) schematic diagram of catalytic mechanisms of L-Ser-CDs and L-Mn-CDs [27].

L-DOPA, an important chiral drug molecule, has been widely used clinically for the treatment of Parkinson’s disease. However, its enantiomer D-DOPA is not only inactive but also exhibits neurotoxic side effects [64]. Carbon dots (CDs) have become a research hotspot due to their easy modification and unique optical properties, and they have been shown to possess a variety of enzyme-like activities, such as SOD, POD, and OXD [65]. There are significant differences in the stereospecific roles of chiral-modified CDs in the regulation of enzyme activity and the mediation of supercoiled DNA topological rearrangements [66]. As shown in Figure 1A, Zhang et al. [26] synthesized the chiral carbon dots (D/L-C-Dots) with OXD-like activity through electrochemical polymerization using L-Ser and D-Ser. The chiral CDs consist of cyclic dipeptide units and possess a hexagonal symmetrical helical dislocation structure. Under the action of CDs, DOPA can be oxidized to form dopachrome, accompanied by the generation of an absorption peak at 475 nm. The catalytic oxidation activity of CDs towards D/L-DOPA was evaluated using UV–Vis analysis, and the results showed that the catalytic activity of L-C-Dots towards L-DOPA was higher than that of D-DOPA, with a selectivity factor of 1.95. However, the D-C-Dots exhibited higher catalytic activity towards D-DOPA than L-DOPA, with a selectivity factor of 2.11. The excellent enantioselectivity is mainly dependent on the hydrogen bonding interactions between L-C-Dots (or D-C-Dots) and L-DOPA (or D-DOPA) being stronger than those between L-C-Dots (or D-C-Dots) and D-DOPA (or L-DOPA). Building upon this work, a recent study utilized Mn-doping to obtain Mn-functionalized CDs (D/L-Mn-CDs), which not only enhanced the POD-like activity of the D/L-Mn-CDs by 6.90-fold but also further improved their enantioselectivity (Figure 1B) [27]. This research provides new insights into enhancing the stereoselectivity of nanozymes.

The stereoselectivity of substrate molecules is realized within specific binding pockets, and amino acids, being the fundamental constituents of most natural enzymes, play a critical role in stereoselective interactions of enzymes with substrates [33]. For instance, Cys has been identified at the active site of natural enzymes that recognize L-DOPA [67]. Utilizing chiral amino acids as ligands to construct stereoselective nanozymes is one of the most commonly used approaches. Qu’s group [23] reported a stereoselective nanozyme with POD-like activity (D/L-Cys@AuNPs-EMSN) that achieves the recognition of DOPA enantiomers. In the prepared nanozyme, expanded mesoporous silica (EMSN) was chosen as the scaffold, AuNPs served as the catalytic active center, and chiral Cys acted as the chiral recognition sites. Molecular dynamics (MD) simulations and apparent steady-state kinetic parameters indicate that L-Cys@AuNPs-EMSN exhibits higher specificity towards L-DOPA, but D-Cys@AuNPs-EMSN displays specificity towards D-DOPA. The primary reason for this specificity lies in the differences in the number of hydrogen bonds formed and π–π interactions between D/L-Cys and D/L-DOPA, respectively. Using the same strategy, another study reported by Qu’s group modified CeNPs with chiral amino acids to obtain a chiral artificial oxidase (CeNP@D/L-Phe), which also demonstrated excellent enantioselectivity in the recognition of DOPA enantiomers [24]. The conjugation of nanomaterials with chiral molecules has stimulated innovative research on the construction of stereoselective nanozymes.

L-Tyr is an essential ketogenic glycogen amino acid and serves as a precursor for neurotransmitters; it is used in regulating mood, treating depression, and alleviating headaches. In contrast to L-Tyr, elevated levels of D-Tyr can serve as a diagnostic indicator for chronic kidney failure [68]. Therefore, accurate detection of Tyr enantiomers holds significant research value in biomedical analysis. Compared to two-dimensional nanomaterials, zero-dimensional quantum dots (QDs) possess more pronounced quantum confinement effects and are more amenable to doping and functionalization. Notably, copper ions (Cu^2+^), which serve as the active sites for different natural enzymes, are often chosen as elements for the design and synthesis of nanozymes [69]. Inspired by this, Zhang et al. [25] prepared chiral quantum dots (D/L-Cys-MoS_2_ QDs) through a top-down synthetic approach, using chiral thiol ligands (Cys) for covalent modification. Under the conjugation of Cu^2+^, D/L-Cys-MoS_2_ QDs/Cu^2+^ exhibit exceptional POD-like activity towards 3,3′,5,5′-tetramethylbenzidine (TMB). To assess the enantioselectivity of chiral QDs towards D/L-Tyr, the absorbance changes at 210 nm were monitored by UV–Vis spectroscopy for Tyr oxidation products. Results indicated that the D-Cys-MoS_2_ QDs/Cu^2+^ displayed significantly higher catalytic activity towards D-Tyr oxidation than L-Cys-MoS_2_ QDs/Cu^2+^. Conversely, the L-Cys-MoS_2_ QDs/Cu^2+^ showed a clear preference for catalyzing L-Tyr oxidation. The enantioselectivity of this chiral QDs is as high as 6.77, which is almost the best performance reported so far. Quartz crystal microbalance (QCM) technology revealed that the enantioselective mechanism arises from differences in the interactions between Tyr enantiomers and chiral QDs. However, it should be noted that further studies are required for a more detailed investigation of the mechanism.

The lack of active sites in nanozymes can hinder their further application in bioanalysis, and the synergistic effect of multiple metals is considered an effective strategy to enhance the catalytic activity of nanozymes [70]. Research has found that horseradish peroxidase (HRP) exhibits stereospecificity towards several enantiomers, such as D/L-DOPA, D/L-Tyr, D/L-tyramine, etc. [71] Hu et al. [46] selected Fe-doped Cu_2−x_Se NPs (Fe_x_Cu_y_Se NPs) and coupled them with D/L-histidine (D/L-His) to develop a highly active and enantioselective nanozyme (D/L-Fe_x_Cu_y_Se NPs) for the recognition of chiral DOPA by mimicking the role of His residues in HRP. Subsequently, a colorimetric sensor was constructed for the detection of L-DOPA in real tablets, achieving a wide detection range (5 μM–0.125 mM, 0.125 mM–1 mM) and a low LOD (1.02 μM), along with good recovery (92.97–107.25%) and relative standard deviations (RSD) of 2.04–9.63%. Ultimately, this method was successfully applied in the quality control of clinical drug L-DOPA.

**Figure 2:**
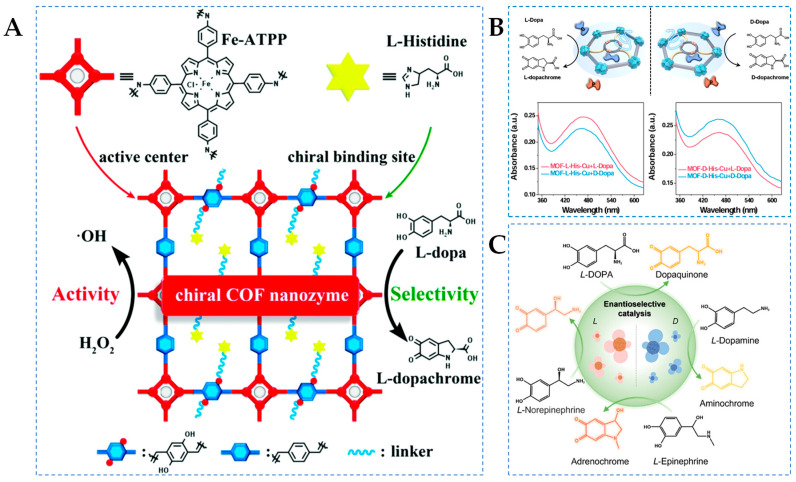
(**A**) Schematic diagram of construction of a chiral COF nanozyme [47], (**B**) schematic of MOF-L(D)-His-Cu-dominated chiral differentiation between the substrate enantiomers [48], and (**C**) schematic diagram of L/D-Pen-nanoflowers mediated catalytic conversion of L-EP, L-DOPA, L-NP, and L-DA [49].

Covalent organic frameworks (COFs) are a newly emerging class of organic porous materials connected by covalent bonds, which can facilitate electron transfer and shorten the electron transfer pathway, making them excellent candidates for achieving high activity [72]. Inspired by the structure of HRP, Zhou et al. [47] synthesized chiral COFs nanozymes (D/L-His@Fe-COF) with iron porphyrin as the active center and D/L-His as the modifying unit (Figure 2A). Experimental results have shown that the stereoselectivity of COFs nanozymes originated from the chiral His unit, which can effectively mimic natural HRP. Additionally, the appropriate modification of L-His imparts specific recognition capabilities to the nanozyme, as well as enhancing its enzyme-like activity, which is 21.7 times higher than that of the natural enzyme. However, the high density of L-His enhances the selectivity factor of COFs nanozymes, and it can also hinder the catalytic oxidation of DOPA. Therefore, the catalytic activity and enantioselectivity of COFs nanozymes can be easily adjusted by changing the doped amino acids and their content. Furthermore, COFs nanozymes exhibit general enantioselectivity for POD-catalyzed chiral reactions, selectively recognizing not only DOPA but also the enantiomers of tyrosinol and Tyr. This work provides a new pathway for the development of highly active stereoselective nanozymes.

Metal–organic frameworks (MOFs), within the realm of crystalline porous materials, are assembled from metals and organic linkers, featuring metal active sites, large surface area, and precisely tailored scaffolds that allow the catalytic and recognition binding units of enzymes to be mirrored in the structure and composition of MOFs through extensive metal–ligand coordination bonds, thereby enhancing enzyme-like catalytic activity and specificity [73]. Natural catechol oxidase (CO) contains a binuclear Cu metal center coordinated by six His residues as active sites, capable of catalyzing the oxidation of catechol [74]. For instance, Sha et al. [48] developed an MOF-L(D)-His-Cu nanozyme using MOF-808 as the basic framework, where chiral His and Cu metal centers were sequentially grafted onto the MOFs to create a catalytic environment and steric hindrance units mimicking COs, thus facilitating the free rotation and conformational adaptation of substrates to provide a basis for achieving high levels of enantioselectivity (Figure 2B). Activation energy measurement experiments demonstrate that the MOF-His-Cu nanozyme can effectively lower energy barriers and exhibit outstanding COs-like activity. Additionally, the UV–Vis spectrum in Figure 2B confirms the enantioselectivity of the MOF-L(D)-His-Cu nanozyme towards D/L-DOPA. The absorbance at 475 nm in the reaction system shows a linear correlation with DOPA concentration (0.6–300 μM), with the LOD of 0.37 μM and 0.50 μM for L-DOPA and D-DOPA based on MOF-L-His-Cu, respectively. The LOD of L-DOPA and D-DOPA are 0.59 μM and 0.51 μM based on MOF-D-His-Cu, respectively. Density functional theory (DFT) reveals that the binding energy and potential spatial effects in the substrate–active site interaction are the reasons for high stereoselectivity.

Interestingly, laccase (LAC), as a multicopper oxidase, can reduce molecular oxygen to produce water, making it an environmentally friendly enzyme [75]. Compared to COs, LAC can oxidize a wide range of phenolic substances and exhibits more significant catalytic capabilities and broader substrate specificity [76]. On the other hand, abnormal neurotransmitter levels can lead to diseases such as schizophrenia, endocrine disorders, and myocardial infarction [77]. In a recent study, Si et al. [49] developed chiral nanoflowers (L/D-Pen-nanoflowers) mimicking LAC activity (Figure 2C). D/L-penicillamine (D/L-Pen) serves as a chiral inducer, coordinating with metal ions to guide the lattice growth direction or induce lattice distortions on the surface of nanostructures, thereby inducing chiral optical activity to inorganic nanostructures. L/D-Pen-nanoflowers exhibit enantioselectivity towards four neurotransmitters, including L-dopamine (L-DA), L-epinephrine (L-EP), L-norepinephrine (L-NE), and L-DOPA. By measuring the surface-enhanced Raman scattering enhancements of L/D-Pen-nanoflowers towards chiral neurotransmitters, it is evident that the differential binding affinities of L/D-Pen-nanoflowers towards chiral neurotransmitters are crucial for chiral recognition. Additionally, the enantioselectivity of natural LAC to D/L-DOPA was measured using UV–Vis spectroscopy and compared with D-Pen-nanoflowers. The calculated selectivity factor of D-Pen-nanoflowers (2.612) is 1.747 times than that of LAC (1.495), demonstrating excellent chiral selectivity.

**Figure 3:**
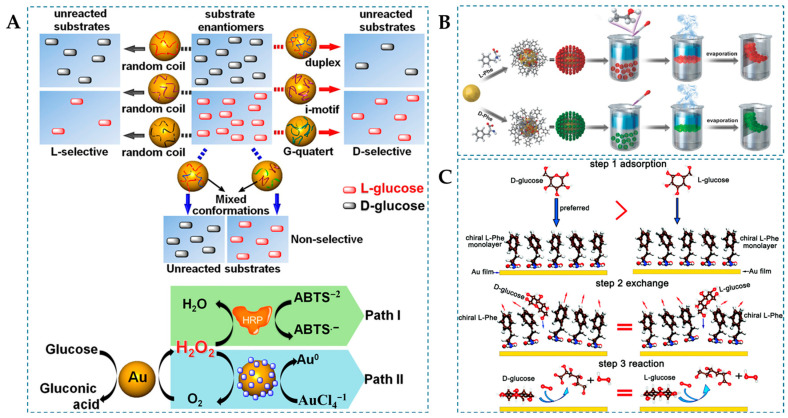
(**A**) Schematic illustration of chiral differentiation between the glucose enantiomers by the catalytic DNA-capped AuNPs [50], (**B**) scheme for the interfacial assembly of the monolayer NP film [51], and (**C**) mechanistic diagram of D/L-Glu catalysis by Pen-capped AuNPs nanoenzymes [85].

D-Glu is directly involved in the human metabolic process and serves as an important biomarker for diagnosing metabolic disorders in diabetic patients [78]. Additionally, D-Glu has teratogenic effects, whereas L-Glu does not, and L-Glu can be taken up by cancer cells for the use in cancer diagnosis [79,80]. Chiral Glu lacks chromophores and is difficult to detect directly via circular dichroism spectroscopy, making the enantioselective recognition of D/L-Glu a continuing challenge [81]. AuNPs are known for mimicking GOx activity that can effectively detect Glu [82]. On the other hand, the unique double helical structure of DNA confers inherent chirality, making it a selector for chiral recognition [83], which can interact with AuNPs to leverage its chemical properties. For instance, Zhang et al. [50] noncovalently adsorbed DNA onto AuNPs to form GOx mimics (Figure 3A), and the interaction between DNA and Glu enantiomers strongly depends on DNA conformation. When subjected to environmental stimuli, randomly coiled DNA-modified AuNPs exhibit higher catalytic activity towards L-Glu than D-Glu, while multichain structures (duplex, i-motif, and G-quadruplex) of DNA molecules reverse the enantioselectivity, favoring the catalysis of D-Glu. The selectivity factor for AuNPs capped with single-stranded DNA (ssDNA) is 1.33, but it is 1.37 for AuNPs capped with double-stranded DNA (dsDNA). The enantioselectivity differences induced by DNA conformation are presumed to be due to the minimization of the benzene-like π-electron surface area by base stacking in randomly coiled DNA, leading to higher affinity with L-Glu. In contrast, structured DNA adopts a configuration with helical minor grooves between the bases, and D-Glu complements the helical grooves of structured DNA better than L-glucose in terms of spatial orientation.

The surface plasmon resonance of AuNPs, as a nanoscale physical phenomenon, presents a potent universal paradigm for enhancing the catalytic performance of nanozymes [84]. As shown in Figure 3B, Xu et al. [51] prepared a single-layer chiral plasmonic film (D/L-Phe-AuNP film) without nanoparticle aggregation, thereby avoiding the limitation of reaction substrate access to catalytic sites caused by nanoparticle aggregation. During the synthesis of the D/L-Phe-NP film, D/L-phenylalanine (D/L-Phe) was introduced for chemical modification. Phe serves as a stabilizer, preventing the formation of irregular multilayer structures of NPs, and as a chiral inducer, imparting plasmonic chirality to the NP film. Under circularly polarized light (CPL) irradiation, the NP film exhibited enhanced GOx-like activity, selectively oxidizing enantiomers of Glu. Furthermore, coupling with HRP can oxidize the colorimetric reagent 2,2′-azino-bis(3-ethylbenzthiazoline-6-sulphonate) (ABTS) to the colored product ABTS^·^^+^ (λ_max_ = 450 nm). This colorimetric reaction allows for spectral analysis of the enantioselectivity of the chiral NP film. Experimental results demonstrate that L-Phe-NP film exhibits higher oxidation activity towards D-Glu, while D-Phe-NP film shows higher oxidation activity towards L-Glu. The catalytic performance of NP films in different CPL irradiation systems also exhibits dependence on left circularly polarized light (LCP) and right circularly polarized light (RCP). LCP significantly enhances the catalytic activity of D-Phe-NP film, while RCP light significantly enhances the catalytic activity of L-Phe-NP film. DFT analysis revealed that the selective interaction of Phe chiral ligands, the increase in steady-state photon intensity, and local field enhancement regulate the stereoselectivity of NP film photocatalysis. In addition, Chen et al. [85] also corroborated, through first-principles calculations, that AuNPs capped with Phe-terminated ligands exhibit stereoselective enzyme-like activity, selectively oxidizing Glu enantiomers, and thereby elucidating the pivotal role of chiral Phe ligands (Figure 3C). This study provides insights into the crucial role of the NP–ligand interface in various applications, inspiring a potential pathway for designing high-performance nanozymes.

To prevent the catalytic sites from being covered, a yolk–shell structure can be utilized to enhance the catalytic activity of nanozymes. Meanwhile, a large number of chiral amino acids can be introduced into the polymer shell to achieve efficient chiral recognition. For example, Zhou et al. [52] utilized yolk–shell-structured magnetite NPs (Fe_3_O_4_ NPs) as the catalytic core within a yolk and a polymer shell appended with chiral tryptophan (D/L-Trp) as the chiral selector, designing a stereoselective nanozyme (Fe_3_O_4_@Poly(Trp)). Fe_3_O_4_@Poly(D-Trp) demonstrated a high selectivity factor of up to 5.38 for D/L-tyrosinol, surpassing even that of the natural HRP of 4.77. Molecular simulations, dialysis experiments, and activation energy studies indicated that the selective adsorption, transport, and desorption of the chiral Trp-appended polymer shell are the reasons for the high enantioselectivity of the nanozyme towards D/L-tyrosinol. Furthermore, Fe_3_O_4_@Poly(Trp) can be used to prepare enantiomerically pure D- or L-enantiomers.

Nanozyme-based colorimetric sensors for chiral recognition mostly rely on the generation of colored products from chiral substrate catalysis by the nanozyme, monitoring the discerning color and changes in UV–Vis absorbance. Ma et al. [53] found that in colorimetric sensors (DCDH@CuNPs) designed for the recognition of chiral DOPA, the introduction of chiral substances can affect the color change induced by the nanozyme-catalyzed chromogenic substrate TMB, which served as the basis for detection. DCDH@CuNPs can oxidize TMB to generate blue-oxidized TMB (oxTMB) in the presence of H_2_O_2_, with a maximum absorption peak at 650 nm. The addition of chiral DOPA enhanced the enzyme-like activity, resulting in an increase in solution color and absorbance. The color and absorbance of DCDH@CuNPs-TMB-H_2_O_2_-D-DOPA were significantly higher than those of DCDH@CuNPs-TMB-H_2_O_2_-L-DOPA, confirming the ability of DCDH@CuNPs to distinguish D/L-DOPA. The enantioselectivity mechanism is attributed to the presence of stronger hydrogen bonding and a higher content of Cu^2+^ (57%) in DCDH@CuNPs-D-DOPA compared to DCDH@CuNPs-L-DOPA, which collectively enhance the catalytic efficiency and lead to a higher production of oxTMB. This study inspires new ideas for designing nanozymes with advanced characteristic oligopeptide ligands. To modulate the catalytic activity and enantioselectivity of nanomaterials, increasing the peptide chain length can be a strategy. For instance, Tian et al. [54] discovered that a tetrapeptide (FFCH) exhibited the highest catalytic activity and best enantioselective recognition ability when modifying CuNPs with a chiral ligand (FFCH@CuNPs). FFCH@CuNPs demonstrated strong hydrogen bonding, π–π interactions, and surface charge with D-DOPA. Furthermore, the two phenyl groups in the FFCH ligand may provide rigid planes that are not easily flipped, further promoting its colorimetric recognition of D/L-DOPA. The developed method showed a linear relationship between absorbance at 650 nm and D-DOPA concentration in the range of 2.0–35.0 μM, with an LOD value of 1.0 μM.

**Figure 4:**
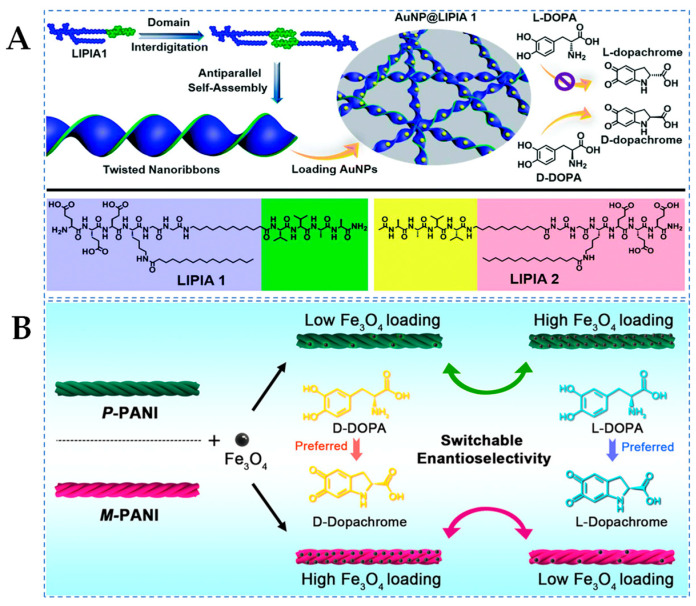
(**A**) Schematic illustration depicting the assembly of supramolecular nanozymes from gold-nanoparticle-decorated twisted nanoribbons and their enantioselective oxidation of D/L-DOPA [29] and (**B**) schematic representation of the enantioselective switchable catalysis of supramolecular chiral PANI-supported magnetic iron oxide nanozymes [41].

The self-assembly of short peptides allows for the creation of twisted nanobelts or helical belts, providing a chiral spatial structure that mimics natural enzymes and creates an environment conducive to enantioselective catalysis [43]. Lai et al. [28] designed hemin and amphiphilic short peptides to self-assemble into helical nanofibers with different chirality. According to the results of UV–Vis spectroscopy analysis, in the catalysis of L-DOPA, the catalytic efficiency of left-handed helical nanozymes (Ac-I_3_*^D^*H-NH_2_) was 1.51 times than that of right-handed helical nanozymes (Ac-I_3_H-NH_2_). Conversely, the catalytic efficiency of right-handed helical nanozymes towards D-DOPA was 1.54 times than that of left-handed helical nanozymes. Molecular self-assembly regulating the arrangement of asymmetric supramolecular scaffolds can aid in constructing stereoselective topological structures as secondary chiral information, providing additional impetus for enhancing the enantioselectivity of nanozymes. As shown in Figure 4A, Yu et al. [29] synthesized sturdy and twisted peptide nanobelts via a peptide crossover mechanism and utilized them as chiral scaffolds for AuNPs, thereby generating supramolecular nanozymes (AuNP@LIPIA 1) that exhibit remarkable catalytic activity and enantioselectivity towards chiral DOPA (selectivity factor of 1.90). This phenomenon strongly depends on the lowered reaction energy barrier and the chiral microenvironment generated by the twisted scaffold. The results indicate that the precise construction of chiral nanostructures through the peptide crossover mechanism, as the chiral matrix of supramolecular nanozymes, possesses improved catalytic performance, promoting the development of advanced biomimetic materials.

The combination of lipophilic ligands and hydrophilic amino acids can be used to synthesize amphiphilic chiral amino acids, thereby allowing for tunable chiral molecules through adjustments in the length of the hydrophobic chain and the hydrophilic head moiety [86,87]. Li et al. [30] constructed chiral supramolecular nanozymes formed by self-assembly of D/L-phenylglycine chiral amphiphilic molecules (D/L-PhgC_16_) and metal ions with POD-like activity. Chiral DOPA was oxidized to generate dopachrome, and by measuring the difference in absorbance at 475 nm, it was found that (*M*)-L-PhgC16-NR-M(ii) selectively oxidized D-DOPA, but (*P*)-D-PhgC_16_-NR-M(ii) exhibited enantioselective preference for L-DOPA, with a selectivity factor of up to 2.80. However, when the alkyl chain length of the phenylglycine amphiphilic molecules was too short, lower rates and negligible enantioselectivity were observed. The synergistic effect between the metal ions and the chiral supramolecular scaffold, along with the chiral transfer between (*P*/*M*)-D/L-PhgC_16_-NR-M(ii) and D/L-DOPA, collectively regulated the enantioselectivity, leading to preferential catalytic oxidation.

Polyaniline (PANI) has been explored for constructing supramolecular chiral structures through various methods, with twisted nanobelts morphology and simple preparation being of particular interest [88]. As shown in Figure 4B, Wang et al. [55] reported a supramolecular chiral nanozyme (*P*/*M*-PANI-Fe_3_O_4_) constructed from nonchiral molecules *P/M*-PANI nanotwists and Fe_3_O_4_ NPs, demonstrating enantioselective catalytic oxidation capability towards D/L-DOPA. Compared to conventional chiral ligands, such as Fe_3_O_4_ nanozymes modified with chiral Phe, it has been elucidated that supramolecular chirality mediated by spatial arrangement has superior enantioselectivity. Interestingly, by adjusting the loading density of catalytic center Fe_3_O_4_ NPs on the chiral PANI, direct control over the accessibility of enantiomers to the catalytic sites on the PANI surface was achieved, leading to unprecedented enantioselective switching during enantioselective catalysis. Furthermore, the research group also constructed supramolecular nanozymes (*P*/*M*-PANI-TA-M^2+^) composed of chiral polyaniline and metal ions (M^2+^, M = Cu, Ni, Co, and Zn) [56]. Through MD simulations, it was once again demonstrated that asymmetric information encoded solely through supramolecular chiral arrangement can provide enantioselective catalytic capability of nanozymes in the absence of chiral molecules, and different metal ions with varying catalytic efficiencies can significantly regulate the overall enantioselectivity, highlighting the synergistic effect between nonchiral ion catalytic centers and chiral supramolecular scaffolds. This study offers profound insights into the roots of asymmetric catalytic mechanisms and propels the design of sophisticated, intelligent nanozymes.

Cyclodextrin (CD) is a cyclic oligosaccharide with multiple chiral centers and a unique hollow structure, comprising hydrophilic outer cavities and hydrophobic inner cavities, enabling CD to play a crucial role in chiral recognition through host–guest interactions [89]. Trp, as an essential amino acid, with L-Trp participating in physiological processes of protein expression, is commonly used to improve mental health disorders. D-Trp has been identified as a precursor for synthesizing anticancer agents and immunosuppressants [90]. Therefore, the recognition and separation of chiral Trp is of utmost importance. Jiang et al. [57] synthesized sulfur quantum dots stabilized by β-CD (CD-SQDs) and developed a fluorescence detection method for chiral Trp. Due to the hydrophobic chiral cavity of β-CD, which resembles natural enzymes, it can form stable host–guest complexes with D/L-Trp. Compared to D-Trp, β-CD more readily forms stable inclusion complexes with L-Trp, and β-CD enzyme mimics can enantioselectively catalyze the hydrolysis of the indole ring of L-Trp. This ultimately led to a significant enhancement in the fluorescence intensity of CD-SQDs upon the addition of L-Trp, but the addition of D-Trp did not. This fluorescence sensing methodology with high sensitivity and specificity had a linear correlation between the fluorescence change of the system and L-Trp concentration in the range of 10–500 nM and an LOD of 2.3 nM. Furthermore, the easy modification and intelligent stimulus responsiveness of CD can expand its application in chiral recognition [91]. Chen et al. [58] covalently attached polycationic a-cyclodextrin α-CD (6-Iz-α-CD) to the surface of AuNPS and synthesized nanozymes (AuNP@6-Iz-α-CD) with GOx-like activity. The catalytic activity of AuNP@6-Iz-α-CD can be modulated by the reversible conformational change upon guest molecule irradiation. Importantly, the chiral cavity of AuNP@6-Iz-α-CD enables enantioselective catalytic oxidation of different configuration of monosaccharides. When combined with the colorimetric reaction of HRP and TMB, the chirality of monosaccharides can be quickly and conveniently observed by the naked eyes. Therefore, intelligent responsive nanozymes provide an unprecedentedly convenient method for colorimetric recognition of chiral monosaccharides.

**Figure 5:**
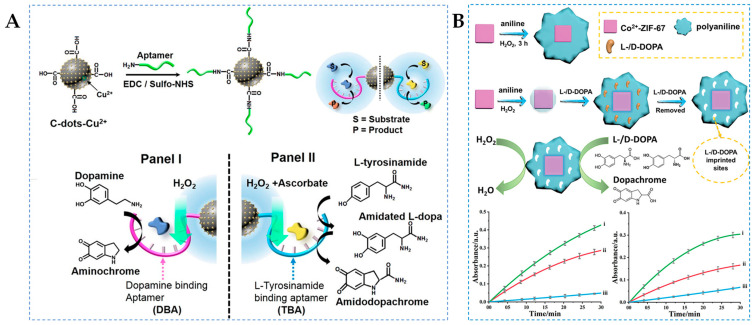
(**A**) Schematic representation of chemical transformations driven by aptananozymes [59] and (**B**) schematic representation of D/L-DOPA imprinted Co^2+^-ZIF-67/PAN for recognition of D/L-DOPA [60].

Compared to natural enzymes, nanozymes lack substrate binding characteristics and concentration of substrate at the catalytic interface. Aptamers are oligonucleotide sequences obtained through systematic evolution of ligands by exponential enrichment technology, which have extremely high specificity and affinity for target substances [92]. Coupling aptamers with nanozymes allows the substrate to be concentrated in the spatial vicinity of the catalytic site, which can enhance the catalytic activity and selectivity for chemical transformation. For example, Ouyang et al. [59] introduced the concept of aptananozymes by conjugating Cu^2+^-functionalized CDs with either the dopamine binding aptamer (DBA) or the L-tyrosinamide binding aptamer (TBA), significantly enhancing the catalytic performance of nanozymes for dopamine and L-tyrosinamide (Figure 5A). In addition to serving as binding sites for substrate concentrations at the catalytic core, the chiral characteristics of aptamer units can induce stereoselective catalysis. UV–Vis spectroscopy analysis indicates that the aptananozymes modified by DBA oxidize L-DOPA approximately twice as much as D-DOPA, and the aptananozymes modified by TBA can selectively oxidize L-tyrosinamide from racemic mixtures. This phenomenon is attributed to the differential binding affinity between the aptamer and chiral substrates, demonstrating that the introduction of aptamers paves the way for obtaining stereoselective nanozymes.

Compared to introducing biological elements, the introduction of molecular imprinting to generate chiral substrate binding sites on nanozymes is a simpler method. The complementary binding cavities in terms of shape, size, and functional groups of the molecular imprinting can accurately recognize chiral molecules, overcoming the drawback of the poor specificity of ordinary nanozymes [93]. As shown in Figure 5B, Wu et al. [60] reported a multimode nanozyme Co^2+^-ZIF-67 NMOFs that can simulate POD, OXD, and CAT activities. Its enzyme-like activity can catalyze the oxidation of aniline to generate polyaniline (PAN) encapsulated nanozymes (Co^2+^-ZIF-67/PAN). After imprinting the chiral reaction site of D/L-DOPA in the polymer coating, the electrostatic interaction, hydrogen bonding, and π–π interaction between chiral DOPA and PAN can serve as the driving force for the imprint cavity recognition. UV–Vis spectroscopy analysis revealed that the catalytic efficiency of D-DOPA-imprinted Co^2+^-ZIF-67/PAN for L-DOPA was 34% lower than that of D-DOPA, and the catalytic efficiency of L-DOPA-imprinted Co^2+^-ZIF-67/PAN for D-DOPA was 47% lower than that of L-DOPA. However, the catalytic efficiency of nonimprinted material was low and it lacked enantioselectivity, confirming that the imprinted molecular site not only provides a molecular pocket for the selective interaction between the substrate and the catalytic site but also introduces an open gate-controlled channel for the interaction between the substrate and the catalytic site.

## 4. Electrochemical Analysis

Electrochemical sensors based on nanozymes have distinct advantages, such as superior selectivity, sensitive detection limits, and ease of miniaturization [94]. Electrochemical sensors for chiral-molecule recognition are mainly based on signal transduction in chiral sensing through ion transport properties. The principle involves nanozyme oxidizing the uncharged ABTS into the positively charged cation radical ABTS^+^ through a cascade reaction, resulting in the changes in ion flux of the system. Therefore, monitoring the changes of ion flux through in situ monitoring of the current–voltage (I–V) characteristics of the sensing system can provide relevant chiral recognition information. The electrochemical analysis for chiral-molecule recognition based on nanozymes is summarized in Table 2.

Clopidogrel (CLP) is a chiral prodrug that undergoes oxidative metabolism through esterase-dependent pathways [104]. S-CLP can effectively inhibit platelet aggregation, but R-CLP is devoid of antithrombotic activity and may cause convulsions in animals at high doses [105,106]. The enzyme-like behaviors of many ester-containing biomolecules have been well reported using natural CD [107]. Upadhyay et al. [95] reported that β-CD is an artificial enzyme model for R/S-CLP recognition on a chiral carbon paste electrode. The complexes formed by β-CD and R/S-CLP, akin to enzyme–substrate complexes, demonstrate different stereospecificity on the carbon paste electrode system. Owing to the steric hindrance and weaker interactions, S-CLP exhibits reduced hydrogen bonding with β-CD compared to R-CLP, leading to a more stable inclusion complex formation between β-CD and R-CLP. Differential pulse voltammetry was used to recognize R/S-CLP, and the peak current showed a linear relationship with R-CLP and S-CLP concentrations in the range of 2.0 × 10^−6^ to 2 × 10^−4^ M, with LOD values of 2.10 × 10^−7^ M and 4.87 × 10^−7^ M, respectively.

**Figure 6:**
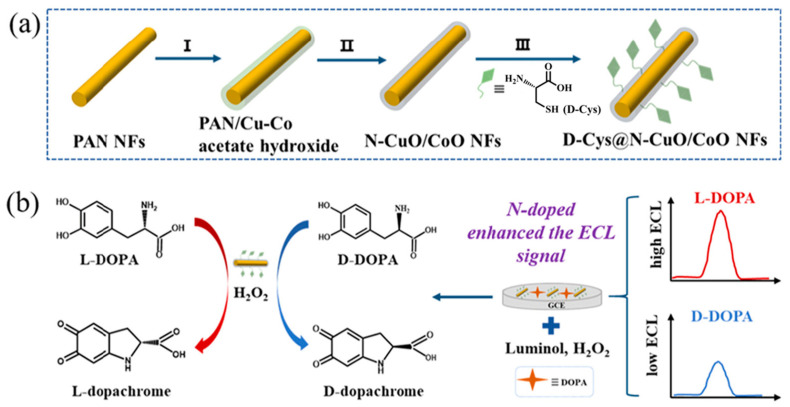
(**A**) Schematic illustration of the synthetic process of D/L-Cys@N-CuO/CoO NFs and (**B**) the enantioselective detection of enantiomers by D/L-Cys@N-CuO/CoO NFs through ECL signals [96].

Electrochemiluminescence (ECL) is a special chemical luminescence phenomenon based on electrochemistry, boasting merits such as a wide dynamic range, high sensitivity, and simple operation [108]. Luminol is one of the most effective and widely used luminescent groups, and it is often applied to construct high-performance electrochemical sensors with nanozymes. For example, Song et al. [96] designed multifunctional nanozymes (D-Cys@N-CuO/CoO NFs) with nitrogen-doped CuO/CoO nanofibers as catalytic active sites and D-Cys as a chiral recognition ligand (Figure 6). The formation of CuO/CoO heterojunctions and nitrogen doping synergistically promoted electron transfer between the nanozyme and the substrate, enhancing the POD-like activity of the nanozyme. In the presence of luminol, D/L-Cys@N-CuO/CoO NFs acted as a sensing platform with significantly enhanced ECL signals, selectively distinguishing and sensitively detecting D-DOPA and L-DOPA. The ECL intensity showed a good linear relationship with the concentration of chiral DOPA in the range of 0.01–0.04 μM, with an LOD of 0.29 nM for L-DOPA and 0.31 nM for D-DOPA. In the detection of fetal bovine serum samples, the spiked recovery of L-DOPA was 94%–103%, with an RSD of less than 5%, attesting to the remarkable proficiency of the developed electrochemical luminescent sensing platform.

Most reported enantioselectivities are achieved by modifying chiral molecules on the surface of nonchiral NPs. In contrast, Au nanocrystals can induce asymmetric structural evolution through chiral ligand induction, thereby generating unique chiral morphologies that can selectively adsorb enantiomers [39]. To enhance the catalytic properties of Au nanocrystals, Liu et al. [97] constructed chiral Au–Pd alloy nanorods (NRs) by synergistically incorporating highly catalytic metal palladium (Pd) and Au through a seed-mediated coreduction growth method. The chiral inducers Cys-Tyr dipeptide (CYP) and Cys were selectively interacted with the NPs surface to form asymmetric facets that led to the formation of chiral crystals at the ends, thereby driving the formation of chiral Au–Pd alloy NRs with two different chiral features (RH/LH-SNRs and RH/LH-HNRs). The NRs exhibited unique enzyme-like activity under CPL excitation and were used to discriminate D/L-Trp using electrochemical methods. LH-HNR showed a higher current response to L-Trp, but RH-HNR showed a higher current response to D-Trp. The difference in binding affinity and adsorption geometry between the surfaces of NRs and chiral Trp can explain this phenomenon. Additionally, the catalytic reaction of chiral Au–Pd alloy NRs with TMB exhibited polarization-dependent nanozyme catalytic activity, revealing a new perspective on controlling nanozymes for tunable optical chiral activity. Wu et al. [109] synthesized intrinsically chiral Au@Pd nanocrystals through a heteroepitaxial growth strategy. Under the optimal conditions, the peak potential difference for oxidation between L-DOPA and D-DOPA was approximately 80 mV, and the oxidation peak current of D-DOPA was twice that of L-DOPA, further demonstrating that integration of catalytic performance and inherent chirality into metal nanocrystals can lead to robust chiral recognition.

**Figure 7:**
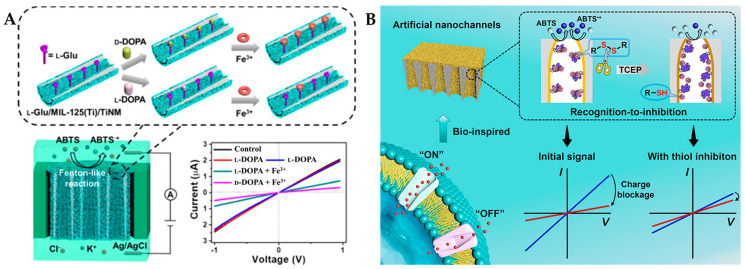
(**A**) Schematic representation of D/L-DOPA recognition by L-glutamine/MIL-125 (Ti) /TiNM nanozymes [98] and (**B**) schematic diagram of the bio-inspired sensing platform for cystine sensing based on a recognition-to-inhibition strategy [99].

In electrochemical sensors, titanium dioxide nanochannel membranes (TiNMs) have garnered significant interest due to their strong chemical stability and inherent photocatalytic activity [110]. TiNMs prepared as homochiral MOF-in-TiNMs electrodes from metal ion precursors exhibited satisfactory recognition performance for chiral DOPA and other enantiomers (Trp, lactate, and carnitine) [111]. As shown in Figure 7A, Dai et al. [98] introduced Fe^3+^ with Fenton-like activity into homochiral nanochannel membranes (L-glutamine/MIL-125(Ti)/TiNM) to design a nanozyme-driven chiral recognition signal amplification strategy. Fe^3+^ formed a high Fenton-like active complex with DOPA through chelation, leading to the oxidation of ABTS to positively charged cationic free radical ABTS^+^, thereby altering the ion transfer flux. Due to the hydrogen bonding interactions between chiral molecules, numerous functional groups on the MOF framework, and smaller nanochannel diameters, D-DOPA was more easily anchored on the nanochannel walls, resulting in greater ion flux changes. The LOD was calculated to be 3.2 μM for L-DOPA and 3.6 μM for D-DOPA based on the I–V curve. Furthermore, L-glutamine/MIL-125(Ti)/TiNM cannot recognize L/D-Tyr, L/D-Trp, and L/D-carnitine, only exhibiting chiral recognition capability for D/L-DOPA with excellent specificity. In addition, the developed method can effectively and dependably detect L-DOPA in genuine sweat samples (recovery of 99.6–107.3%). This study provides inspiration for developing asymmetric nanochannels for recognizing chiral molecules.

Cystine, as a dimeric amino acid derivative, is a component of hair and nail keratin [112]. Cystinuria is a disorder characterized by impaired transport of cystine and its oxidized dimer, resulting in high concentrations of L-cystine in human urine, highlighting the importance of chiral recognition of cystine [113]. As shown in Figure 7B, Xu et al. [99] constructed a chiral nanochannel membrane (Fe^3+^: L-MOFs/TiNM) for the recognition of D/L-cystine. Through an innovative signal transduction strategy based on recognition-to-inhibition, tris(2-carboxyethyl) phosphine hydrochloride (TCEP) induced the cleavage of disulfide bonds in cystine, leading to the formation of HS-tails that block the catalytic site (Fe^3+^) of the nanozyme, which can inhibit its activity and modulate the I–V characteristics. L-tartaric acid (L-TA) served as a chiral recognition selector that can selectively capture L-cystine over D-cystine through configuration-dependent hydrogen bonding selectivity. As a result, the chiral sensor exhibited a high discrimination sensitivity towards L-cystine, in the concentration range of 0.025–0.4 mM with an LOD of 13.6 μM.

The orderly biochemical reactions within living organisms mainly rely on the high efficiency and specificity of natural enzymes for catalysis. For instance, L-lactate dehydrogenase selectively oxidizes L-lactate, while D-lactate dehydrogenase oxidizes D-lactate [114]. Dopamine-β-hydroxylase inserts a hydroxyl group into the β-carbon on the side chain of dopamine, stereoselectively producing an R-enantiomer [115]. In the presence of molecular oxygen, GOx specifically oxidizes β-D-Glu to generate gluconic acid and H_2_O_2_, remaining chemically inert towards β-L-Glu [116]. The conjugation of natural enzymes with nanozymes not only retains the high stereoselectivity of natural enzymes but also capitalizes on the high catalytic activity and stability characteristic of nanozymes.

Inspired by the natural mineralization of wood, Dai et al. [100] grew Prussian blue (PB) within the wood channels using a diffusion technique, which possesses POD-like activity, and then combined it with GOx through electrostatic interactions. This mild method of enzyme immobilization did not disrupt the native conformation of the enzyme, endowing the wood channels with the capability to recognize the enantiomers of Glu. In this sensing platform (GOx/PB/CM), the specificity of natural enzyme GOx catalyzes the oxidation of D-Glu to produce H_2_O_2_ and gluconic acid in the presence of dissolved O_2_, leading to a decrease in pH value and activating PB’s POD-like activity to catalyze the generation of positively charged ABTS^+^. Benefiting from the confinement effect of the channel and the asymmetric mineralization of PB, a significant change in transmembrane ion flux occurs. Conversely, as GOx cannot catalyze the oxidation of L-Glu, these cascade reactions are inhibited. In situ monitoring of ion transport behavior through I–V characteristics revealed a larger ion current response when D-Glu and ABTS coexisted. The dual-enzyme system (GOx/PB) can generate a significant amount of ABTS^+^, which is crucial for chiral Glu recognition. Additionally, the pronounced differences in I–V characteristics provided a reliable strategy for L/D-Glu recognition. GOx/PB/CM exhibited good linear responses to D-Glu within the concentration ranges of 0.01–1 mM and 1–10 mM, with an LOD of 3 μM. The recognition platform demonstrated high selectivity and good reproducibility, as evidenced in the detection of real samples such as blood, urine, and sweat.

The combination of nanozymes with natural enzymes and the host–guest interactions between nanozymes and chiral substances provide a new approach for designing efficient stereoselective nanozymes. MOFs not only possess multiple mimetic enzyme activities but also serve as perfect scaffolds for immobilized enzymes, overcoming the fragility of natural enzymes, and can achieve cascade reactions of natural enzyme nanozymes [117]. Zhang et al. [101] utilized chiral Ni-MOFs as nanozymes and carriers to immobilize acetylcholinesterase (AChE) for the synergistic catalysis of acetylthiocholine chloride (ATCl) and efficient detection of the chiral drug inhibitor galantamine (GH). Different pore-sized chiral Ni-MOFs (D-Ni-PYR, D/L-Ni-BPY, and D-Ni-BPB) exhibited significant synergistic catalytic effects with AChE towards ATCl, and the L/D-Ni-BPY system demonstrated stronger host–guest interactions with ATCl and higher catalytic efficiency. Furthermore, monitoring the relationship between oxidation current and GH concentration revealed a linear correlation in the concentration range of 1 × 10^−12^ M to 1 × 10^−6^ M, with LODs of 8.09 pM, 0.51 pM, 0.31 pM, and 1.69 pM for the D-Ni-PYR, D-Ni-BPY, L-Ni-BPY, and D-Ni-BPB sensing systems, respectively. Microcalorimetric studies indicated that the chiral environment between L-Ni-BPY and chiral GH matched better, leading to a stronger chiral interaction and enhanced inhibition by attracting more GH molecules, facilitating their diffusion into the MOFs pores for efficient inhibition. Therefore, matched pore sizes and chirality not only enhance the interactions between substrates in MOFs pores and active sites, but also maximize the changes in enzyme catalytic effects, improving the performance of the sensing system. Additionally, the sensing system exhibited good selectivity and stability, enabling effective detection of GH in human serum samples with satisfactory accuracy (recovery of 97.00%–105.22%).

**Figure 8:**
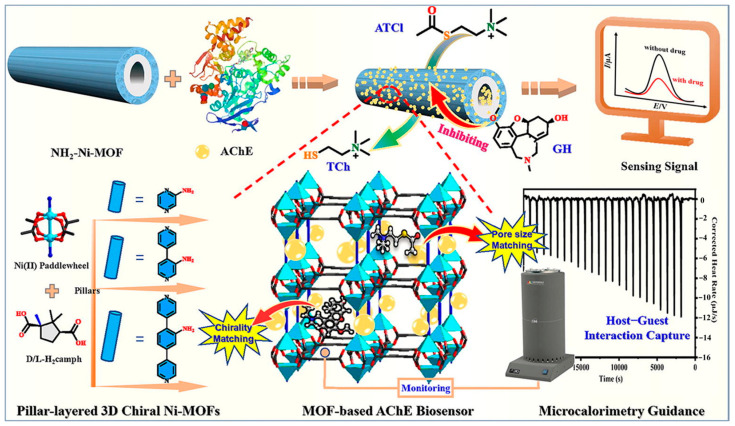
Schematic diagram of the preparation and sensing performance of the Ni-MOF-based AChE biosensor, showing the microcalorimetry-guided microenvironment optimization of MOF pore [101].

The substitution of natural enzymes with nanozymes for constructing nanozyme-nanozyme cascades eradicates the reliance on natural enzymes, offering enhanced stability and cost-effectiveness. Mesoporous MOFs avoid the limitation of active site access caused by microporous structures, enhancing the accessibility and catalytic efficiency of MOFs [118]. As shown in Figure 8, Guo et al. [102] synthesized asymmetric mesoporous homochiral CuMOF nanochannels (Meso-L-CuMOF/TiO_2_M) and filled AuNPs into the mesoporous pockets of Meso-L-CuMOF through in situ reduction, integrating the enzyme-mimicking of AuNPs and Fenton-like activity of CuMOF in a cascading reaction to design a target-induced signal sensing strategy for chiral Glu recognition and quantification. During localized surface plasmon resonance excitation, the nanoconfinement effects of the dielectric CuMOF substrate and mesoporous framework facilitated the generation of high-energy charge carriers, enhancing the enzyme-mimicking and Fenton-like activities. Numerical simulations and experimental results demonstrated that the chiral environment induced by L-CuMOF made D-Glu more easily oxidized than L-Glu, producing more ABTS^·^^+^ and causing a significant increase in ion current. Through sensitive signal transduction provided by I–V characteristics, D-Glu was quantitatively recognized within two linear ranges of 0.1–1 μM and 1.0–10 μM, with an LOD of 0.089 μM.

D-Pen is an essential therapeutic agent for treating diseases such as Wilson’s disease, hepatic renal fibrosis, and rheumatoid arthritis [119]. However, L-Pen can cause olfactory degradation and neuropathy due to its toxicity [120]. Xu et al. [103] developed a sensing device MnO_2_/L-MIL-125/TiNM for recognizing D/L-Pen. The sensing device consisted of a chiral recognition area with L-TA grafted TiNM nanochannels and a reaction area modified with MnO_2_ at the tip of TiNM. MnO_2_ exhibits multienzyme activities (GOx and POD activities) and can execute different catalytic functions through self-cascading synergistic effects, simplifying the system design. Due to the strong affinity between L-Pen and L-TA, D-Pen exhibits a weaker affinity for L-TA. More L-Pen molecules are anchored inside the nanochannels, but L-Pen molecules can easily pass through the recognition area and reach the reaction zone. The D-Pen molecules trigger the reduction of MnO_2_ to Mn^2+^, diminishing the cascade enzyme-like activity of MnO_2_. Therefore, the oxidation of ABTS is hindered, leading to a decrease in ionic current. By recording the change in ion current, D-Pen exhibited a linear response in the concentration in the range of 0.05–1.0 mM, and the LOD value was 0.013 M. The recovery rates of D-Pen in real water samples were in the range of 98.8–104.0%, indicating the potential applicability and reliability of the sensor. The research lays groundwork for the development of multifunctional, enantiomer-specific nanozyme-based fluid sensors, making the recognition of chiral molecules possible.

## 5. Discussion and Prospects

Chirality constitutes a fundamental attribute of biological systems, and chiral recognition holds significant importance in various fields. Chiral nanozymes ingeniously combine the catalytic specificity of enzymes with the unique properties of nanomaterials, making them excellent candidates for highly sensitive detection and chiral recognition of chiral molecules in biological and environmental samples. Although researchers have made outstanding progress in the study of chiral nanozymes, the exploration of these nanozymes is still in its early stages, with many shortcomings and significant room for improvement. In the future, research on stereoselective recognition of chiral molecules based on nanozymes needs to consider some challenges and obstacles.

(1) Insufficiently detailed chiral recognition mechanisms. Despite thermodynamic parameters, activation energy calculations, and molecular simulations demonstrating the crucial role of interactions (such as hydrogen bonding, π–π interactions), chiral microenvironments, and nanoconfinement effects in chiral-molecule recognition, there are still chiral nanozymes with unclear recognition mechanisms. Some nanozymes exhibit preferences for homochirality, while others show preferences for heterochirality. Therefore, further exploration is necessary to elucidate more detailed and universally applicable chiral recognition mechanisms in nanozymes.

(2) Limited range of chiral recognition targets. Most chiral molecules recognized based on nanozyme activity in the existing reports are D/L-DOPA and chiral Glu. Although there has been some research on chiral amino acids, there is a lack of recognition for chiral agrochemicals (insecticides, herbicides, etc.) and chiral drugs (e.g., naproxen, ibuprofen, and tramadol). Additionally, the recognition and separation of geometric isomers and analogs with similar physicochemical properties are crucial. Therefore, the development of multifunctional nanozymes to broaden their application scope is necessary.

(3) There are limited fluorescent sensors available for chiral recognition within nanozymes. Fluorescence techniques excel at offering both high selectivity and high-throughput analysis capabilities, enabling swift in situ recognition of chiral compounds. Chiral AIEgens display remarkable luminescence when aggregated, with selectivity reaching over 16,800 [121]. Constructing fluorescence nanozymes based on chiral AIEgens can achieve chiral-molecule recognition. Furthermore, integrating multiple detection modes, including fluorescence, colorimetry, and electrochemical analysis, can effectively mitigate background interference, thereby ensuring more dependable analytical outcomes.

(4) Lower catalytic activity and stereoselectivity of most nanozymes compared to natural enzymes. When constructing chiral sensors based on nanozymes, it is essential to consider the factors that contribute to high stereospecificity. The enantioselectivity of most nanozymes comes from the modification of chiral ligands (amino acids, DNA), mimicking the primary structure of natural enzymes. However, surface modifications may hinder the catalytic sites of nanozymes, leading to a selectivity factor generally lower than two. Inspired by the spatial structure of natural enzymes, supramolecular chiral scaffolds, molecular imprinting, and chiral MOF nanochannels establish conducive microenvironments for enantioselective catalysis, transcending reliance on chiral center atoms alone. Additionally, plasmonic chiral nanocrystals, besides intrinsic chirality, exhibit polarization-dependent enantiomeric selectivity and plasmon-enhanced nanozyme catalytic activity under CPL excitation, providing effective tools for developing highly catalytic and specific nanozymes.

(5) Interactions in chiral recognition are easily influenced by pH, temperature, and ion strength conditions. As an illustration, high temperature can disrupt hydrogen bonds, thus hindering the chiral recognition of nanozymes. Nonetheless, chiral recognition based on nanozymes rarely considers operation under unfavorable conditions. Consequently, future research needs to consider the impact of environmental factors on chiral recognition and develop high-performance chiral nanozymes to overcome existing deficiencies and better serve as natural enzyme alternatives.

## 6. Conclusions

This review comprehensively summarizes the research progress in enantioselective catalysis and the recognition of chiral compounds, facilitated by electrochemical techniques and UV–Vis spectroscopy based on nanozymes. Carbon materials, metal nanoparticles, metal oxides, MOFs, etc., have been successfully designed as enantioselective nanozymes for chiral-molecule recognition. In recent years, strategies to enhance the enantiomer selectivity of nanozymes have involved the application of chiral ligand modifications, the induction of supramolecular chiral scaffolds, and the exploitation of pore confinement effects in homochiral MOFs, as well as the introduction of molecular imprinting and aptamers. To enhance catalytic activity, strategies such as metal doping, cascading natural enzymes and nanozymes, and self-cascading of multienzyme active nanomaterials have been utilized. However, further exploration is needed for multi-signal and multimode chiral recognition, a wider range of recognition targets, and detailed recognition mechanisms. This review aims to provide a new perspective on the advancement of chiral nanozymes, attracting more researchers to delve deeper into research and develop highly active and specific nanozymes.

## Figures and Tables

**Table 1:** Summary of UV–Vis spectroscopy analysis for chiral-molecule recognition based on nanozymes.

Nanozyme	Activity	Analyte	Method	LOD (μM)	Linear Range (μM)	Selectivity Factor	Ref.
S-CuO	POD	R/S-MA	UV–Vis	–	–	–	[21]
D-Cys@Au/Fe Hops	POD	D-DOPA	UV–Vis	–	–	3.70	[40]
L-Cys@Au/Fe Hops		L-DOPA		–	–	2.81	
Fe-P-GO-COOH/Fe-M-GO-COOH	POD	D/L-DOPA	UV–Vis	–	–	–	[45]
D-C-Dots	OXD	D/L-DOPA	UV–Vis	–	–	2.11	[26]
L-C-Dots						1.95	
D/L-Mn-CDs	OXD	D/L-DOPA	UV–Vis	–	–	–	[27]
D-Cys@AuNPs-EMSN	POD	D/L-DOPA	UV–Vis	–	–	1.47	[23]
L-Cys@AuNPs-EMSN						1.69	
CeNP@D-Phe	OXD	D/L-DOPA	UV–Vis	–	–	1.13	[24]
CeNP@L-Phe						1.87	
D/L-Cys-MoS_2_ QDs	POD	D/L-Tyr	UV–Vis	–	–	6.77	[25]
D-Fe_x_Cu_y_Se NPs	POD	D/L-DOPA	UV–Vis	1.02 (L)	5–125, 125–1000	1.49	[46]
L-Fe_x_Cu_y_Se NPs						1.56	
D-His@Fe-COF	POD	D/L-DOPA	UV–Vis	–	–	1.51	[47]
L-His@Fe-COF						1.86	
MOF-D-His-Cu	COs	D/L-DOPA	UV–Vis	0.37 (L); 0.50 (D)	0.6–300	2.12	[48]
MOF-L-His-Cu				0.59 (L); 0.51 (D)		2.19	
D/L-Pen-nanoflowers	LAC	L-EP	UV–Vis	–	–	1.96	[49]
		L-DA				1.61	
		L-NE				1.83	
		L-DOPA				2.97	
DNA-capped AuNPs	GOx	D/L-Glu	UV–Vis	–	–	1.33; 1.37	[50]
D/L-Phe-NP film	OXD	D/L-Glu	UV–Vis	–	–	–	[51]
Fe_3_O_4_@Poly(D-Trp)	POD	D/L-Tyrosinol	UV–Vis	–	–	5.38	[52]
Fe_3_O_4_@Poly(L-Trp)						4.02	
DCDH@CuNPs	POD	D/L-DOPA	UV–Vis	–	–	1.85	[53]
FFCH@CuNPs	POD	D/L-DOPA	UV–Vis	1.0 (D)	2.0–35.0	–	[54]
Ac-I_3_H-NH_2_, Ac-I_3_*^D^*H-NH_2_	POD	D/L-DOPA	UV–Vis	–	–	–	[28]
AuNP@LIPIA 1	POD	D/L-DOPA	UV–Vis	–	–	1.90	[29]
(*M*)-L-PhgC_16_-NR-M(ii)	POD	D/L-DOPA	UV–Vis	–	–	2.35	[30]
(*P*)-D-PhgC_16_-NR-M(ii)						2.80	
*P*-PANI–Fe_3_O_4_	POD	D/L-DOPA	UV–Vis	–	–	1.98	[55]
*M*-PANI–Fe_3_O_4_						1.88	
*M*-PANI-TA-M^2+^	POD	D/L-DOPA	UV–Vis	–	–	1.70	[56]
*P*-PANI-TA-M^2+^						2.07	
CD-SQDs	hydrolase	D/L-Trp	Fluorescence	2.3 × 10^−3^ (L)	0.01–0.50	–	[57]
AuNP@6-Iz-α-CD	GOx	D/L-Ribose, D/L-Lyxose, D/L-Xylose, D/L-Mannose, D/L-Glu	UV–Vis	–	–	–	[58]
Aptamer-Modified Cu^2+^-Functionalized CDs	Aptananozymes	D/L-DOPA	UV–Vis	–	–	4.20	[59]
Co^2+^-ZIF-67/PAN	POD	D/L-DOPA	UV–Vis	–	–	–	[60]

POD, peroxidase; OXD, oxidase; CAT, catalase; COs, catechol oxidase; GOx, glucose oxidase; LAC, laccase; MOFs, metal–organic frameworks; R/S-MA, R/S-mandelic acid; D/L-DOPA, D/L-3,4-dihydroxy-phenylalanine; GO-COOH, carboxyl-modified graphene oxide; Fe-*P*/*M*, right/left-handed spiral zinc-finger-protein like α-helical supramolecular complex; D/L-C-Dots, D/L- serine-Carbon dots; CDs, carbon dots; D/L-cysteine; EMSN, expanded mesoporous silica; D/L-Cys, D/L-Cysteine; QDs, quantum dots; D/L-Tyr, D/L-Tyrosine; NPs, nanoparticles; COFs, covalent organic frameworks; D/L-Phe, D/L-phenylalanine; L-DA, L-dopamine; L-EP, L-epinephrine; L-NE, L-norepinephrine; D/L-His, D/L-histidine; DCDH, D-cysteine-D-histidine; FFCH, L-phenylalanine-L-phenylalanine-L-Cysteine-L-Histidine; LIPIAs, lipid-inspired peptide-interdigitating amphiphiles; D/L-Glu, D/L-glucose D/L-Trp, D-/L-tryptophan; D/L-Pen, D/L-penicillamine; *M*/*P* NRs, helical nanoribbons; L/D-PhgC_16_, L/D-phenylglycine chiral amphiphiles; PANI, Polyaniline; M^2+^, M = Cu, Ni, Co, and Zn; TA, thioglycolic acid; CD, cyclodextrin; SQDs, sulfur quantum dots; 6-Iz-α-CD, polycationic a-cyclodextrin; PAN, polyaniline; ZIF-67, zeolitic imidazolate framework-67. “–” means not provided.

**Table 2:** Summary of electrochemical analysis for chiral-molecule recognition based on nanozymes.

Nanozyme	Activity	Analyte	Method	LOD (μM)	Linear Range (μM)	Selectivity Factor	Ref.
OPECT	OXD	D/L-Glu	Electrochemical	0.05 (D); 0.07 (L)	0.1–10, 10–10,000	–	[22]
GNS-PNP-β-CD-CPE	esterase	R/S-CLP	Electrochemical	0.210 (S); 0.487 (R)	2.0–200	–	[95]
D-Cys@N-CuO/CoO NFs	POD	D/L-DOPA	Electrochemical	3.10 × 10^−4^ (D); 2.90 × 10^−4^ (L)	1 × 10^−2^–4 × 10^−2^	1.36	[96]
L-Cys@N-CuO/CoO NFs						1.71	
LH/RH-SNRS, LH/RH-HNRS	POD	D/L-Trp	Electrochemical	–	–	–	[97]
L-glutamine/MIL-125 (Ti) /TiNM	POD	D/L-DOPA	Electrochemical	3.2 (L); 3.6 (D)	10–200, 200–1000	–	[98]
Fe^3+^: L-MOFs/TiNM	POD	D/L-Cystine	Electrochemical	13.6 (L)	25–400	–	[99]
GOx/PB/CM	POD	D/L-Glu	Electrochemical	–	–	–	[100]
D-Ni-PYR, D-Ni-BPY, L-Ni-BPY, D-Ni-BPB	OXD	GH	Electrochemical	8.09 × 10^−6^; 5.1 × 10^−7^; 3.1 × 10^−7^; 1.69 × 10^−6^	1 × 10^−6^–1	–	[101]
Au@Meso-L-CuMOF/TiO_2_M	POD, OXD	D/L-mannose, D/L-xylose, D/L -tagatose, D/L-ribose, D/L-galactose, D/L-Glu	Electrochemical	0.089 (D-Glu)	0.1–1; 1.0–10	–	[102]
MnO_2_/L-MIL-125/TiNM	GOx, POD	D/L-Pen	Electrochemical	13 (D)	50–1000 (L); 25–1000 (D)	–	[103]

POD, peroxidase; OXD, oxidase; GOx, glucose oxidase; OPECT, organic photoelectrochemical transistor; D/L-DOPA, D/L-3,4-dihydroxy-phenylalanine; D/L-Glu, D/L-glucose; R/S-CLP, Clopidogrel; GNS, graphene; PNP, platinum nanoparticle; β-CD, β-cyclodextrin; NFs, nanofibers; CPE, carbon paste electrode; RH, right-handed; LH, left-handed; SNRS, spiral nanorods; HNRS, helical nanorods; NRs, nanorods; TiNM, TiO_2_ nanochannel membrane; PB, Prussian blue; CM, cellulosic membrane; GH, galanthamine; AChE, acetylcholinesterase; PYR, 2-aminopyrazine; BPY, 3-amino-4,4′-bipyridine; BPB, 2,5-di(pyridine-4-yl) aniline; TiO_2_M, TiO_2_ nanochannel membrane; Meso, mesoporous; D/L-Pen, D/L-penicillamine. “–” means not provided.

## Data Availability

Not applicable.
